# Teleporting into walls? The irrelevance of the physical world in embodied perspective-taking

**DOI:** 10.3758/s13423-022-02070-8

**Published:** 2022-12-07

**Authors:** Steven Samuel, Sarah Salo, Tiia Ladvelin, Geoff G. Cole, Madeline J. Eacott

**Affiliations:** 1 School of Psychology, University of Plymouth, Portland Square, Drake Circus, Plymouth, PL4 8AA UK; 2 Department of Psychology, University of Essex, Colchester, UK

**Keywords:** Visual perspective-taking, Level 2 perspective-taking, Embodied cognition, Mental rotation

## Abstract

Embodied theories of Level 2 visual perspective-taking usually assume that we imagine ourselves in other real-world locations to take perspectives. We tested this assertion by giving participants an embodied perspective-taking task in which they were instructed to make manual responses based on imagined perspectives. Importantly, on half of the trials, the location of the alternative perspective was not physically possible (i.e., blocked with a wall). Across two experiments, results showed that participants performed just as well for the physically impossible perspectives as for accessible ones. We interpret these data as evidence that embodied perspective-taking is agnostic to local physical features of our environment.

**Supplementary Information:**

The online version contains supplementary material available at 10.3758/s13423-022-02070-8.

Visual perspective-taking (VPT) is considered crucial to our ability to understand, communicate, and deal with other agents (Brown-Schmidt et al., 2008; Clark & Brennan, 1991; Linde & Labov, 1975). The question of *how* we manage this task has been the subject of much research, and we have learned, for example, that there is a distinction between the ability to understand simply *whether* something is seen (Level 1 VPT), and the later-developing understanding that some things can appear different depending on viewpoint (Level 2 VPT: Flavell et al., 1981; Masangkay et al., 1974; Sodian et al., 2007). Level 2 VPT, being less susceptible to nonperspectival alternatives such as geometric line-of-sight reasoning (Michelon & Zacks, 2006), is believed to be a better candidate if the aim is to investigate perspective-taking as about others’ mental states (Lurz, 2009).

One way in which Level 2 VPT problems are solved concerns the imagined relocation of our physical selves to the required perspective location, variously called *embodied perspective-taking, grounded perspective-taking* or *viewer rotation* (e.g., Cavallo et al., 2017; Erle & Topolinski, 2017; Surtees et al., 2013). For example, to understand what is on the left or right of someone facing us, we might imagine ourselves standing at their location, allowing us to make our judgments pseudo-egocentrically. Evidence for embodied VPT comes from performance impairments when participants’ bodies are physically rotated or fixed in a posture incongruent with the shortest path to an imagined location (Deroualle et al., 2015; Kessler & Rutherford, 2010; Kessler & Thomson, 2010; Surtees et al., 2013; Yu & Zacks, 2017). Participants have also been shown to experience difficulty judging what is on another person’s right or left when doing so requires imagining a body part unusually rotated (Parsons, 1987), or imagining body configurations which violate norms of articulation (Amorim et al., 2006). When tasked with locating a target in a grid from another perspective but making a manual response (button press) spatially mapped to their own perspective, participants sometimes press the button that would be correct from the alternative perspective instead (Samuel et al., 2019). In terms of theory, embodied perspective-taking also fits well within the broader context of embodied cognition, which posits a role for motor representations underlying various cognitive processes that do not lead to actual physical movement (Barsalou, 2008; Pulvermüller, 2005), as well as simulation-based accounts of mental state reasoning (Gallese & Goldman, 1998; Gordon, 1986).

The evidence for embodied VPT is thus compelling, but while the evidence for the role of the perspective-taker’s body is substantial, evidence for any role of the physical world in which perspective-taking takes place is lacking. If embodied VPT concerns the relocation of the self to other *real-world locations*, then we should expect physical impediments in our environment (e.g., walls) to have the same negative impact as more direct interference with our bodies, even though we do not experience contact with them at the crucial moment. An alternative view is that we perform a more limited process by which we imagine ourselves in a new location relative to a target object or scene *without* representing features of our environment (i.e., in an imagined space akin to a blank page). This latter process would suggest a more cognitively efficient but also less informationally rich representation.

There is some support for the imagined space possibility. Samuel et al. (2019) instructed participants to discriminate target digits (e.g., a 6) from distractor digits (e.g., a 9) from an avatar’s perspective, but to press a button mapped to the target’s spatial location from their own perspective. Both the target and distractor were located in a 2 × 2 grid, displayed on a screen laid flat in front of the participant, with each grid square spatially mapped to four buttons in front of the participant. The avatar could appear at any of the four edges around the display, and the target and distractor were always upright from the avatar’s perspective. This meant if that avatar was at a 90-degree angle to the participant on the right of the display (for example) and saw the target in the top left square, the participant should press the *bottom* left button, as this corresponded to the target’s location from their own point of view. However, participants sometimes pressed the button corresponding to the avatar’s perspective instead (e.g., the top left), which corresponded to an empty square from their own perspective. Importantly, they did this at greater than chance levels (i.e., fewer or no responses at all were made to the only other empty square in the grid).

Samuel and colleagues interpreted these ‘altercentric responses’ as evidence of the integration of motor representations with the avatar’s perspective to locate the target, and the subsequent failure to disengage from this reference frame prior to making a manual response. In a second experiment, the avatar was replaced with a black circle and participants located targets assuming the circle indicated the true bottom of the screen. Despite this instruction now favouring the mental rotation of the grid rather than the relocation of the participant, the pattern of altercentric errors was repeated. Samuel and colleagues suggested that the integration of one’s motor representations with an imagined scene can occur whether imagining movement of the self *or* the target object/scene.

In the present experiment, we tested whether the imagined relocation of the self is crucial to embodiment more directly. We adapted the task used by Samuel et al. (2019) with the central exception that the participant and the computer display were always placed against a wall on one side, making it physically impossible to move to that side of the display. If embodied VPT occurs in real space, performance should be impaired when the desired location is out of bounds. If embodiment is a more limited process consisting of just the self and the target object/scene in neutral space, it should be possible to make responses equally well from possible and impossible locations.

We made two other important changes, both of which were designed to prime embodied processes as strongly as possible. Firstly, participants were instructed not only to locate the target from the avatar’s perspective but also to make a physical response consistent with it. In other words, what previously would have been considered an altercentric error would in the present experiment be a correct response. Secondly, participants were instructed to use their left hand to press the left two buttons and their right hand to use the right two buttons, for all perspectives. In the original version, the dominant hand was used for all response buttons. This change also promoted embodiment, as it required participants to sometimes use their left hand to respond to something on their own right, and vice-versa, in accordance with the perspective location they were adopting.

## Experiment 1

### Method

Details of the preregistration of the methods and analyses for this study can be found here (https://osf.io/a7tb4).

#### Participants

A power calculation using G*Power software found that 35 participants were required for an 80% chance to detect a medium effect size (difference in RT between physically *possible* and *impossible* trials, based on a two-tailed paired-sample *t* test with alpha = .05, *d* = 0.5, correlation between measures = .5). We considered a medium effect size the minimum of interest because Samuel et al. (2019) had found a large effect size (*r* = .575) when comparing the number of altercentric errors to control errors.^1^ All participants were required to demonstrate a minimum 70% accuracy across the task as a whole (where chance is 25%). A total of nine participants were replaced for not reaching this threshold (range: 25%–50%), demonstrating evidence that they pressed the key that corresponded to the location of the target from their own viewpoint instead of the avatar’s. The final sample therefore comprised 36 participants (*M*_age_ = 19 years, range: 18–22, males = 11). All were required to have normal or corrected-to-normal vision and hearing and to speak English as a first language (for the most automatic possible comprehension of the auditory stimuli). All were recruited using the University of Essex online recruiting system and were compensated with course credit. Total participation time was approximately 30 minutes.

#### Materials and procedure

**Fig. 1:**
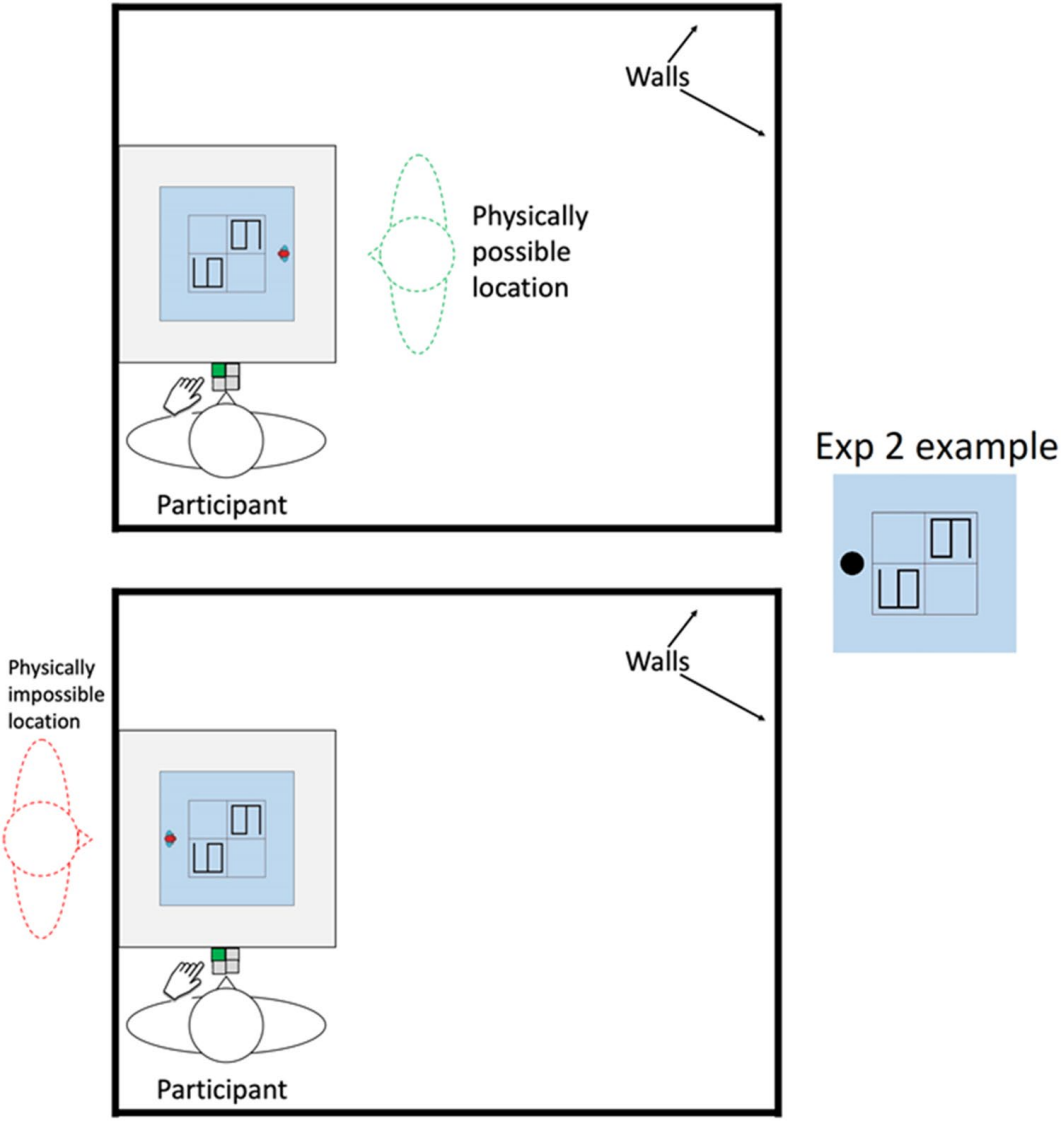
Top-down schematic of the lab. The examples come from a *related* trial (distractor is the target rotated 180 degrees) with the target 6 from the avatar’s perspective, from Experiment 1. In the top picture the participant could imagine their body on the same side of the display as the avatar (physically *possible* trial), but in the bottom picture there is no room owing to the wall (physically *impossible* trial). The correct answer in both cases is the top left button, with the left forefinger. In Experiment 2, the avatar was replaced with a filled black circle marking the bottom of the screen

Participants were told that they would hear a number and then be shown a 2 × 2 grid on a computer with two numbers, each in a separate square in the grid (see Fig. 1). They were instructed to locate the target number from the avatar’s perspective and press a button on the button box that corresponded to the target’s location from that point of view. For example, the avatar might call a ‘six’ and then appear on the right of the grid. The participant should then find the 6 the avatar is referring to, which in this case would look the participant would look like a 6 lying on its side. If that target is in the top left corner of the grid from the avatar’s perspective, the correct answer would be the top left button.

Participants sat at one edge of a computer screen laid flat on a table. The button box was placed between the participant and the display. A white cardboard border attached to the screen created a square 720^2^ pixel/16 cm^2^ window in which the grids were displayed, to minimize any sense of a ‘correct’ viewing angle of the screen. Each trial began with an instruction (1,000 ms), which was always either ‘six’ or ‘four’ spoken in a female voice (the avatar was described as female). The screen was blank during the instruction and for 250 ms afterwards. An empty grid then appeared (100 ms duration), followed immediately by the two digits—one the target, one a distractor—always in diagonally opposed squares. At the same time, the avatar appeared on either the bottom, left, top, or right of the grid. Only the avatar’s head (covered with a red cap), shoulders, and ends of her shoes were visible. The target (either 4 or 6) was always upright from the avatar’s perspective. The trial terminated when the participant responded, or after 3,500 ms had elapsed (a time-out). There was then 2,000 ms of blank screen prior to the next trial.

All instructions were printed on paper. Prior to the experiment proper there was a brief (12-trial) practice session with ‘+’ as the target to train participants in the two-handed manual response mode. Participants were instructed to use their left forefinger for the two left-sided buttons, and their right forefinger for the two right-sided buttons.^2^ Participants then received some example grids printed on paper and were required to show the experimenter which button on the response box they would press. If the participant did not display the correct choices, or did not use the correct hand, further examples and clarification were provided until the participant did.

On half the trials the distractor digit was the target rotated 180^o^ (*related* condition). This was to minimize the chance of participants relying on digit form alone (instead of perspective) to disambiguate the target. On the other half the distractor was a different digit—namely, a 4 if the target was a 6, and vice-versa (*unrelated* condition). The avatar appeared at each of the four sides of the grid 25% of the time (shared perspective, left perspective, opposite perspective, right perspective). Overall, therefore, the 64 experimental trials of each block were equally divided between *related/unrelated* trials, ‘four’/’six’ targets, target and competitor location, and avatar position.

Half (*n* = 18) of the participants performed the first block with the wall to their left, the other half (*n* = 18) with the wall to their right. The relevant edge of the screen border was always touching the wall, and the participant could not move in the direction of the wall. Participants left the room for a moment while the computer was moved across the room between blocks. Each block consisted of 64 randomly presented trials, 16 of which therefore required a physically impossible perspective and response. Block order was counterbalanced across participants.

Our primary analysis was the comparison between RTs on physically Possible and Impossible trials, collapsed over side (left/right), by means of a paired-sample *t* test. In the case of null results, we required that the data be at least three times more probable under the null than the alternative hypothesis (Dienes, 2014).

### Results

#### Physically impossible perspective locations

**Fig. 2:**
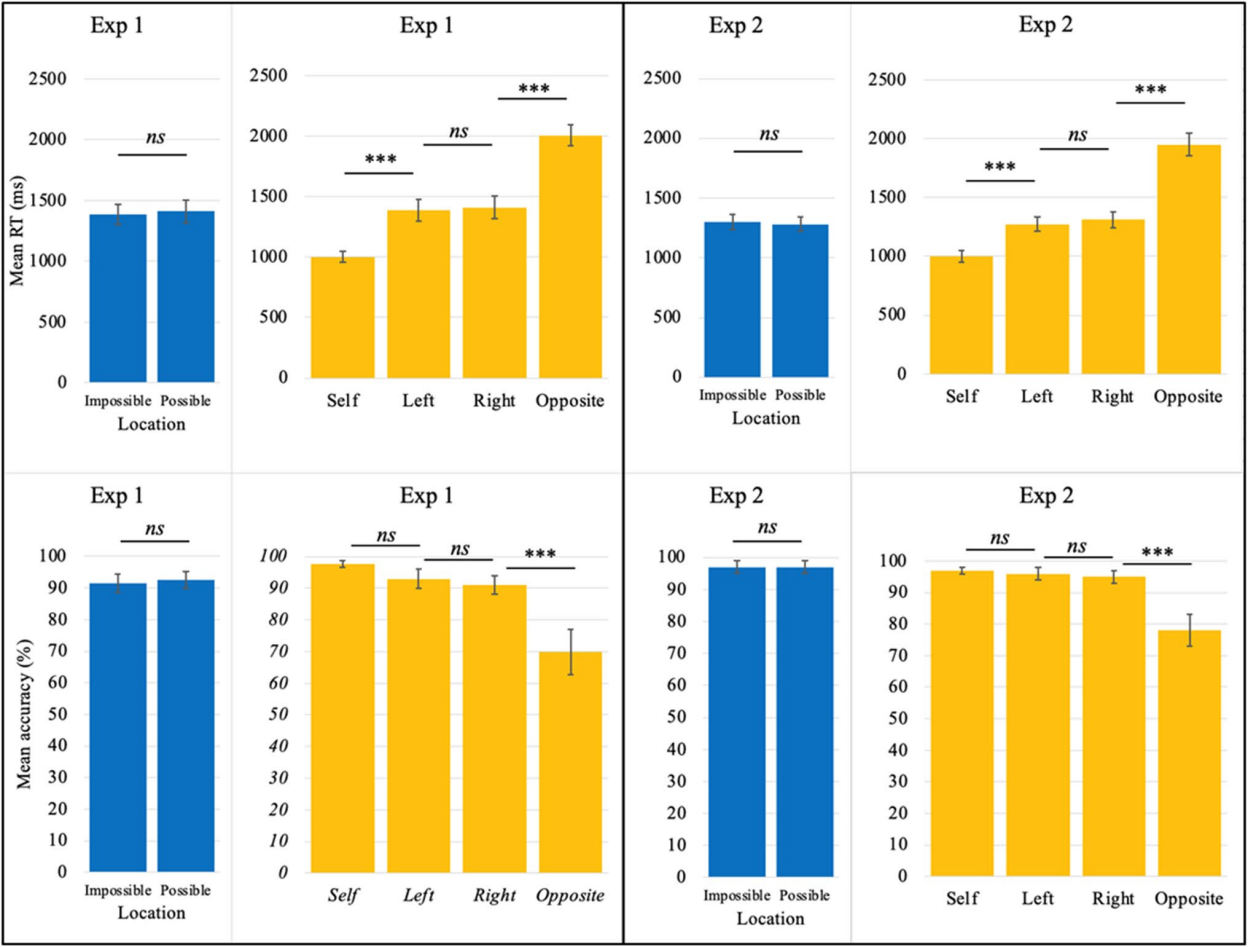
Results. Error bars represent 95% confidence intervals. Only a subset of pairwise comparisons is shown here (those which allow most other comparisons to be inferred); full details can be found in the text

Initial analyses found that the distribution of mean RTs on trials with *impossible* and *possible* trials did not deviate from normality (Shapiro–Wilk tests, all *p*s > .29). A paired-sample *t* test found no statistically significant difference between the two variables, *M*_Diff_ = 25 ms, 95% CI_Diff_ [−66, +16], *t*(35) = 1.229, *p* = .227, *d* = 0.205 [−0.127, 0.534] (see Fig. 2, top-left panel). A Bayesian test found that the data were 11 times more likely under the null that physically impossible perspectives do not lead to slower performance (BF_10_ = 0.087). Accuracy was high overall (*Mdn*_Possible_ = 94%, *Mdn*_Impossible_ = 94%). A Wilcoxon related-samples signed rank test found no statistically significant difference between possible and impossible trials, *W* = 211, *p* = .361 (see Fig. 2, bottom-left panel).

#### Other results

We also compared mean RTs across all four perspectives to check that performance on the task met the expectation that it was easier to respond according to one’s own perspective than alternative viewpoints, particularly opposite-perspective trials. Initial analyses found that the distributions of the four relevant RT variables did not deviate from normality (Shapiro–Wilk tests, all *p*s > .18). A repeated-measures ANOVA using the Greenhouse–Geisser correction for violation of sphericity found the expected main effect of perspective, *F*(2.25, 78.736) = 259.829, *p* < .001, η_p_^2^ = .881 (see Fig. 2, upper center-left panel). As predicted, all contrasts were significant at the *p* < .001 level with the exception of left vs. right (*p* = 1), in every case Bonferroni-corrected for multiple comparisons. Accuracy across the task as a whole was high (*M* = 88%, 95% CI [85%, 91%], range 70%–100%). A related-samples Friedman’s ANOVA by ranks found an effect of perspective in accuracy, chi square = 72.866, *p* < .001 (see Fig. 2, lower center-left panel), with all contrasts significant at the *p* < .01 level (Bonferroni-corrected) with the exception of left vs. right and left vs. self (*p* = 1 and *p* = .120, respectively).

#### Post hoc tests

We conducted two unregistered analyses. First, we investigated whether there was an effect of the wall early on which faded with practice. A 2: Order (Block 1 vs. Block 2) × 2 Space: (Possible vs. Impossible) fully within-participants ANOVA found a main effect of Order, *F*(1, 35) = 81.281, *p* < .001, η_p_^2^ = .549, with responses on Block 2 on average 299 ms faster than on Block 1, 95% CI [232, 367]. There was no main effect of Space, *F*(1,35) = 1.279, *p* = .266, η_p_^2^ = .003, and crucially no interaction, *F*(1, 35) = 0.234, *p* = .632, η_p_^2^ = .001. Second, we examined whether responses that would be made with the same hand from the egocentric perspective were faster than responses that would be made with a different hand, which would add support for transformations of the egocentric frame of reference. This ruled out analyses of opposite-perspective trials, which always reversed hands, leaving left- and right-perspective trials. A 2: Order (Block 1 vs. Block 2) × 2: Hand (Same vs. Different) × 2 Space: (Possible vs. Impossible) fully within-participants ANOVA revealed the main effect of order found previously, *F*(1, 35) = 78.084, *p* < .001, η_p_^2^ = .372, and also a main effect of hand, *F*(1,35) = 19.347, *p* < .001, η_p_^2^ = .047. Overlapping responses were indeed faster than nonoverlapping responses, *M*_Diff_ = 107 ms, 95% CI [57, 156]. No other main effects nor interactions reached significance (all *p*s > .06).

### Discussion

Results from Experiment 1 showed that accuracy was high and performance similar across both possible and impossible perspectives. Our data therefore support the view that embodied VPT is agnostic to the physical features of the environment in which the task takes place.

An alternative view, however, is that participants may have represented themselves within the virtual space in our experiment, enabling the correct manual responses to be made without disruption from the real physical wall. Previous research has shown that participants can imagine themselves within virtual spaces. Cavallo et al. (2017) showed participants computer-generated images of a room and asked them to indicate the location an apple on a table from either their own perspective or the other side of the table. They found that participants were faster to indicate that the apple was on their right than on their left from the self-perspective *and* from an opposite perspective (i.e., when the apple was actually on their left), an effect they interpreted as evidence of embodiment through the transferal of participants’ right-side dominance (e.g., through right-handedness) even to imagined perspective locations. In support of this view, the researchers also found that this right-sided advantage disappeared on opposite-perspective trials if the required perspective location was made physically inaccessible through the deployment of barriers either side of the table. The authors argued that the barriers made the participants change strategy, mapping the apple onto a neutral space rather than imagining themselves at the other side of the table. 

It is important to note that, given contemporary accounts of embodiment make clear that it is a process influenced by physical realities, ‘entering’ a 2D representation of a 3D world would also violate this assumption. Nevertheless, our task may have failed to test our hypothesis about real-world barriers because participants could imagine themselves in the virtual space occupied by the avatar, where there were no barriers to movement.

## Experiment 2

### Method

In Experiment 2 we minimized the chances that participants could conceive of the stimulus as an inhabitable virtual space, while maximizing the chances that they imagined themselves in other locations in the real world. Following a procedure used by Samuel et al. (2019), we replaced the avatar with a filled black circle, which we explained was a marker for the bottom of the screen. Replacing the avatar in this way meant that the stimulus was now a flat image, also lacking a humanoid figure to embody. We also explicitly instructed participants that the aim of the task was ‘to indicate where in the grid the number is *as if you were sitting at the edge of the table that corresponds to the bottom of the grid*’ (italics in original). Previous research with these same stimuli and with a similar instruction found that participants made altercentric errors consistent with an embodiment strategy (Samuel et al., 2019). Experiment 2 was otherwise identical to Experiment 1, except where stated. Details of the pre-registration for this study can be found here (https://osf.io/5v98n).

#### Participants

The sample comprised 36 participants (*M*_age_ = 20 years, range: 18–29, males = seven, females = 28, Nonbinary = one). All were recruited using the University of Plymouth online recruiting system and were compensated with course credit.

#### Materials and procedure

The original E-Prime experiment was converted for use in Open Sesame, with all the same stimuli, sound files, and timings. Participants performed 12 practice trials based on the experimental task, with feedback, rather than locating and responding to ‘+’ signs in the grid. This was to reduce the number of replacements for low accuracy. In Experiment 2, only two participants were replaced for this reason. The practice trials were performed in the same location as the first block. The viewing window in the screen overlay was increased slightly, from 16 cm^2^ to 18 cm^2^.

### Results

#### Physically impossible perspective locations

There were no significant deviations from normality (Shapiro–Wilk tests, all *p*s > .49). There was no statistically significant difference between possible and impossible perspectives, *M*_Diff_ = 18 ms, 95% CI_Diff_ [−6, 41], *t*(35) = 1.514, *p* = .139, *d* = .25 [−0.08, 0.58] (see Fig. 2, upper center-right panel), and the data were approximately twice as likely under the null (BF_10_ = 0.507). Accuracy was high overall from left-sided and right-sided perspectives (both medians = 97%), with no statistically significant difference between impossible trials and possible trials, *W* = 111, *p* = .167 (see Fig. 2, lower center-right panel).

#### Other results

As before, we also compared mean RTs across all four perspectives. Three of the relevant RT variables did not deviate from normality (Shapiro–Wilk tests, all *p*s > .5), one did (Shared Perspective; *p* = .03). A repeated-measures ANOVA for all four perspectives found the expected main effect, *F*(1.808, 63.266) = 304.992, *p* < .001, η_p_^2^ = .897 (see Fig. 2, top right panel), with all contrasts were significant at the *p* < .001 level with the exception of left vs. right (*p* = 1), Bonferroni-corrected. To verify that the nonnormal distribution of Shared Perspective data did not skew results, unplanned Wilcoxon tests were run on contrasts using these data, which confirmed the differences (*p*s < .001). Accuracy across the task as a whole was high (*M* = 92%, 95% CI [90%, 94%], range: 72%–100%). A related-samples Friedman’s ANOVA by ranks found an effect of Perspective in accuracy, chi square = 59.913, *p* < .001 (see Fig. 2, bottom right panel), with all contrasts significant at the *p* < .01 level (Bonferroni-corrected) with the exception of Shared vs. Left (*p* = .28), Shared vs. Right (*p* = .73), and Left vs. Right (*p* = 1).

#### Post hoc tests

Shapiro–Wilk tests found no evidence of deviations from normality from any cell (all *p*s > .27). A 2: Order (Block 1 vs. Block 2) × 2 Space: (Possible vs. Impossible) fully within-participants ANOVA found a main effect of Order, *F*(1, 35) = 7.979, *p* = .008, η_p_^2^ = .105, with responses on Block 2 on average 77 ms faster than on Block 1, 95% CI [22, 133]. There was no main effect of Space, *F*(1, 35) = 2.234, *p* = .144, η_p_^2^ = .005, and no interaction, *F*(1, 35) = 0.139, *p* = .712, η_p_^2^ = .001. A 2: Order (Block 1 vs. Block 2) × 2: Hand (Same vs. Different) × 2 Space: (Possible vs. Impossible) fully within-participants ANOVA revealed main effects of Hand, *F*(1,35) = 42.050, *p* < .001, η_p_^2^ = .081, and Order, *F*(1,35) = 7.801, *p* = .008, η_p_^2^ = .055. Responses were faster on Block 2 than Block 1, by 76 ms, 95% CI [21, 131], and overlapping responses were faster, *M*_Diff_ = 92 ms, 95% CI [63, 121]. No other main effects nor interactions reached significance (all *p*s > .16).

### Discussion

The results of Experiment 2 replicated those of Experiment 1; participants performed as quickly and as accurately when the desired perspective location was obstructed by a wall as when there were no impediments. Although the observed data were twice rather than three times more likely under the null hypothesis (our preset threshold) they still favored the null, making the difference only a matter of degree.

## General discussion

We gave adults a task in which they were required to make a manual response consistent with one of four possible perspectives. In Experiment 1, adults were as accurate and efficient at adopting physically impossible perspectives as those which were unimpeded. This result was replicated in Experiment 2, where we minimized the possibility that participants may have been imagining themselves within the target scene. Together, these findings suggest that adults have no difficulty taking physically impossible perspectives.

Our results support a form of embodied perspective-taking that is agnostic to the physical environment. However, Cavallo et al. (2017) found that participants’ usual response-time advantage locating objects on their right translated to the opposite perspectives only if it was physically accessible. Participants’ performance was compared across two conditions; one where participants responded according to the perspective of an avatar, the other from an impossible location flanked by two wardrobes. Thus, the two conditions differed by accessibility but also by avatar presence and the presence of two new objects. It is difficult to speculate as to how (and whether) one of these other variables may have given rise to those results. Here, the only difference was accessibility, and we used real rather than computer-generated barriers. We also required a manual response that was mapped both horizontally and vertically to the target perspective (participants in Cavallo et al., 2017, responded verbally). We feel the current design is therefore better placed to test the notion of embodiment as a physical process. It is important to note that Cavallo and colleagues also found no significant difference in RTs between the avatar and inaccessible conditions.^3^ In this sense our results are consistent with theirs.

Embodied perspective-taking has traditionally been described as imagined relocation, but an embodiment account need not posit that the perspective taker imagines *moving* somewhere else, only that they imagine *being* somewhere else relative to the target scene. We feel our results are best explained by the latter. By this account, the reason we found no effect of the wall is not because participants can imagine themselves ‘within’ the wall, but because the representation generated is created ‘from scratch,’ populated only with those elements fundamental to making manual responses consistent with other points of view, which in the present experiments was the grid and its new (imagined) spatial orientation relative to the self. This is more parsimonious than an alternative account by which the environment perhaps *is* represented but only abstractly, shorn of its physical properties. This account also allows embodiment effects to arise even as the result of representations generated through array rotations. For example, participants could have imagined an array rotation which integrated motor representations with the scene in the same way as imagining self-movement, because the representation generated via either processes should be identical (Samuel et al., 2019).

Our post hoc analyses showed that responses were faster when the same hand would have been used from both the egocentric and imagined perspective. This is consistent with the possibility that participants imagined themselves in a new physical relationship relative to the grid, benefitting from any overlap with their egocentric frame of reference. However, this is also consistent with the fact that stimulus–response conflict requires more processing (May & Wendt, 2012, 2013). The danger therefore is that there is no clear evidence that our participants were embodying perspectives and thus it is understandable that the walls had no effect. We feel this is highly unlikely for a number of reasons. First, the stimulus–response compatibility alternative is more neatly applied to purely spatial tasks, such as judging whether an object is in an agent’s left or right hand, but not when identifying a perspective-contingent property of that object, which was necessary to identify the target in the first place. Any spatial compatibility effects are therefore the *outcome* of perspective-taking in this paradigm, not an explanation of it. However, it is likely that compatibility effects also contribute to the increase in response times through increasing angles of disparity (as incompatibility increases). Importantly, other studies using this paradigm have shown that performance is affected by factors entirely unrelated to compatibility, such as slower responses the more closely the distractor resembles the target (Samuel et al., 2019) and when the target’s identity is misleading rather than simply ambiguous (Samuel et al., 2020). In sum, there is information crucial to the task that can only be gleaned through perspective-taking, and while we do not claim that all participants used embodiment all the time (indeed, we have just speculated that it is possible to see embodiment effects even following mental rotation of a scene rather than the viewer), we do not feel that stimulus–response compatibility is a viable alternative account of task performance here.

In conclusion, embodied perspective-taking appears to involve a narrower and specific representation concerning the self and the target alone, rather than the imagined relocation of the self to other real-world locations. This allows for manipulations that directly impact the body influence the efficiency of perspective-taking as they would also influence real physical movement. However, effects that the surrounding environment *would* have upon the body if it moved, such as physical barriers, show no such effect.

## Supplementary Information

ESM 1 (DOCX 18 kb)

ESM 2 (CSV 7 kb)

ESM 3 (CSV 9 kb)

## Footnotes

1 Altercentric errors are responses to an empty square from the participant’s perspective. On each trial there was one other empty square that did not contain the target from either perspective, the selection of which represented a control error.

2 A different study (not reported here) found a tendency for there to be more altercentric responses when manual responses were lateralised, presumably because doing so boosts the strength of the integration of one’s own left and right with the imagined perspective.

3 We are grateful to the authors for providing their data, which allowed us to confirm this.
