# Supplementary material for: Teleporting into walls? The irrelevance of the physical world in embodied perspective-taking
Source: Psychon Bull Rev. 2022 Dec 7;30(3):1011–9. doi: 10.3758/s13423-022-02070-8 (PMC10264460; doi:10.3758/s13423-022-02070-8)
Supplement: Supplementary file 1 — (DOCX 18 kb) [file 13423_2022_2070_MOESM1_ESM.docx]

Data processing info

To create Same and Different hand data:

Number pad arrangement:

89

56

thus:

*When perspective is LEFT:*

keys 6 and 8 are consistent (same hand)

*When perspective is RIGHT:*

keys 5 and 9 are consistent (same hand)
